# Targeted Metabolomics Analysis of Bile Acids in Patients with Idiosyncratic Drug-Induced Liver Injury

**DOI:** 10.3390/metabo11120852

**Published:** 2021-12-08

**Authors:** Zhongyang Xie, Lingjian Zhang, Ermei Chen, Juan Lu, Lanlan Xiao, Qiuhong Liu, Danhua Zhu, Fen Zhang, Xiaowei Xu, Lanjuan Li

**Affiliations:** 1 State Key Laboratory for Diagnosis and Treatment of Infectious Diseases, National Clinical Research Center for Infectious Diseases, Collaborative Innovation Center for Diagnosis and Treatment of Infectious Diseases, The First Affiliated Hospital, College of Medicine, Zhejiang University, Hangzhou 310009, China; zyxie@zju.edu.cn (Z.X.); 11918236@zju.edu.cn (L.Z.); lujuanzju@zju.edu.cn (J.L.); lanlan.xiao@zju.edu.cn (L.X.); liuqh@zju.edu.cn (Q.L.); zdhla@126.cn (D.Z.); 11818053@zju.edu.cn (F.Z.); 2 Department of Gastroenterology, Zhongshan Hospital Affiliated to Xiamen University, Xiamen 361004, China; cemsmiles@163.com

**Keywords:** drug-induced liver injury, metabolomics, bile acid, severity

## Abstract

Drug-induced liver injury (DILI) is rare but clinically important due to a high rate of mortality. However, specific biomarkers for diagnosing and predicting the severity and prognosis of DILI are lacking. Here, we used targeted metabolomics to identify and quantify specific types of bile acids that can predict the severity of DILI. A total of 161 DILI patients were enrolled in this prospective cohort study, as well as 31 health controls. A targeted metabolomics method was used to identify 24 types of bile acids. DILI patients were divided into mild, moderate, and severe groups according to disease severity. A multivariate analysis was performed to identify characteristic bile acids. Then the patients were divided into severe and non-severe groups, and logistic regression was used to identify bile acids that could predict DILI severity. Among the enrolled DILI patients, 32 were in the mild group, 90 were in the moderate group, and 39 were in the severe group. Orthogonal partial least squares-discriminant analysis (OPLS-DA) modeling clearly discriminated among the different groups. Among the four groups, glycochenodeoxycholate (GCDCA), taurochenodeoxycholate (TCDCA), deoxycholic acid (DCA), Nor Cholic acid (NorCA), glycocholic acid (GCA), and taurocholic acid (TCA) showed significant differences in concentration between at least two groups. NorCA, GCDCA, and TCDCA were all independent risk factors that differentiated severe DILI patients from the other groups. The area under the receiver operating characteristic curve (AUROC) of GCDCA, TCDCA, and NorCA was 0.856, 0.792, and 0.753, respectively. Together, these three bile acids had an AUROC of 0.895 for predicting severe DILI patients. DILI patients with different disease severities have specific bile acid metabolomics. NorCA, GCDTA, and TCDCA were independent risk factors for differentiating severe DILI patients from less-severe patients and have the potential to predict DILI severity.

## 1. Introduction

Drug-induced liver injury (DILI) is a rare but clinically important entity that can be induced by small chemical molecules, biological agents, traditional Chinese medicines (TCMs), natural medicines (NMs), health products (HPs), and dietary supplements (DSs) [1]. The incidence of DILI is 2.3 to 40.6 per 100,000 people in Western countries [2]. In China, the incidence is 23.8 per 100,000 people [3]. DILI remains a major cause of acute liver failure (ALF) in Western countries [4] and poses a 10% mortality risk in the United States [5]. Meanwhile, approximately 5.7% to 39% of patients will develop chronic DILI, which is defined as persistent liver function abnormalities for at least 6 months [6].

Idiosyncratic (unpredictable) DILI is one of the most challenging liver disorders, because of the myriad of drugs used in clinical practice, available herbs and dietary supplements (HDS) with hepatotoxic potential [7]. Although DILI has received increasing attention, the pathogenesis is complex and remains unclear. It also likely differs among subjects for a given drug [2]. Recent studies have elucidated that a combination of various host, drug, and environmental factors determines susceptibility to DILI and its phenotypic expression [8]. Yet, specific biomarkers critical for diagnosing and predicting the severity and prognosis of DILI are still lacking.

The liver is central to the biotransformation (metabolism) of xenobiotics and endogenous substances. DILI causes huge changes in the metabolism of related substances, which can be detected by metabolomic approaches. Metabolomic approaches enable the analysis of disease-related changes in the metabolome and facilitate the identification of therapeutic targets and biomarkers [9]. A previous study [10] that used untargeted metabolomic detection technology revealed that the metabolome was abruptly changed during DILI progression and that the primary bile acid biosynthesis pathway was correlated with the severity of DILI, which might play a vital role in disease progression. However, the specific types and concentrations of bile acids need to be confirmed by targeted metabolomic techniques due to limited detection using untargeted approaches. 

In this study, targeted ultra-performance liquid chromatography tandem mass spectrometry (UPLC/MS) was used to quantify 24 types of bile acid in sera of idiosyncratic DILI patients. We explored the changes in DILI and analyzed the relationship between bile acids and DILI severity. Then, several specific bile acids were screened to identify whether they could be used to predict DILI severity. 

## 2. Results

### 2.1. Clinical Characteristics of Enrolled Patients

A total of 161 DILI patients and 31 healthy controls were enrolled. In all, 32 patients had a disease severity of Grade 1 (mild), 90 had Grade 2 (moderate), and 39 had Grade 3 (severe) DILI. The demographic, biochemical, and clinical characteristics are summarized in Table 1 and Supplementary Table S1. The suspected drugs causing DILI are listed in Supplementary Table S2. 

The age and sex were comparable between different groups, and female patients accounted for most cases (68.3%). The mean age of DILI patients was 51 years, and there were no differences among mild, moderate, and severe groups (*p* = 0.937). Most patients presented with liver dysfunction within 5 to 90 days (71.9% in mild, 84.4% in moderate, and 87% in severe groups, *p* = 0.179). In the moderate group, TBA (*p* < 0.001), GGT (*p* < 0.01), TB (*p* < 0.001), TG (*p* < 0.001), VLDL (*p* < 0.001), and INR (*p* < 0.05) were significantly higher compared to the mild DILI group, while ALB (*p* < 0.001), HDL (*p* < 0.001), and LDL (*p* < 0.001) were significantly lower. In the severe group, WBC (*p* < 0.01) and INR (*p* < 0.001) were much higher than those in the moderate group, while ALB (*p* < 0.001), ALP (*p* < 0.001), GGT (*p* < 0.001), TG (*p* < 0.001), cholesterol (*p* < 0.001), and VLDL (*p* < 0.001) were lower. Interestingly, GGT, TG, and VLDL showed inconsistent trends during disease progression, which increased in the moderate group and then dropped in the severe group.

According to the R value, the most common pattern of liver injury was hepatocellular in all three groups (81.3% in mild, 63.3% in moderate, and 64.1% in the severe group). All patients had an RUCAM score > 3, and most patients had a score between 6 and 8.

### 2.2. Multivariate Analysis of Targeted Metabolomics Data of Bile Acids

**Figure 1:**
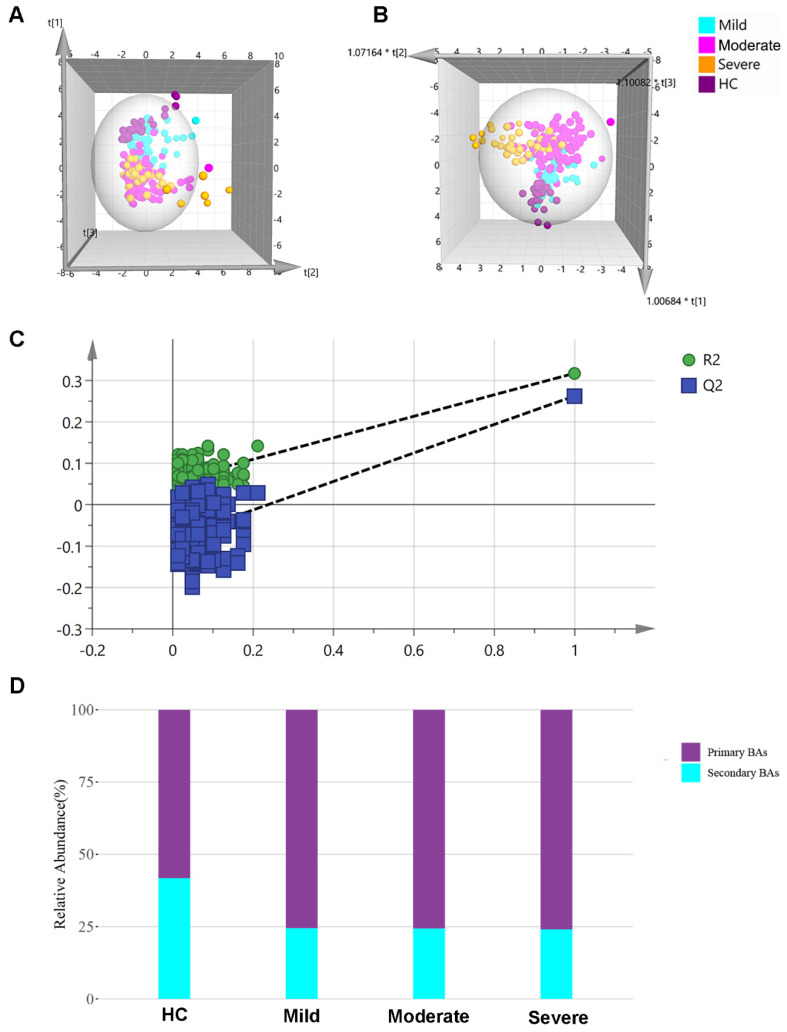
Metabolomic profiling by targeted metabolomics. (**A**) PCA−X and (**B**) OPLS−DA 3D models among HC, mild, moderate, and severe groups. (**C**) The 200−permutation test demonstrated no overfitting in the PLS-DA model [Q2 = (0.0, –0.119)]. (**D**) Proportion of primary and secondary bile acids in different groups. HC: healthy control, PCA: principal component analysis, OPLS-DA: orthogonal partial least squares-discriminant analysis.

A total of 24 different types of bile acid (detailed in Supplementary Table S3) were detected and precisely quantified in the samples. The concentration levels of 24 bile acids in different groups were shown in Supplementary Table S4, and they were all imported into SIMCA-P+ software for multivariate analysis, as shown in the 3D PCA and OPLS-DA scatter plot (Figure 1A,B). HC and DILI samples were well separated, and plots from different groups were clustered according to the OPLS-DA model. In order to detect whether the supervised pattern analysis method has overfitting phenomenon, the PLS-DA model needs to be tested. Generally, a 200-permutation test is selected, and if the final ordinate of Q2 is less than 0.05, it indicates that the model has no overfitting and the model is reliable. The 200-permutation test of our data demonstrated no overfitting in the PLS-DA model [Q2 = (0.0, −0.119)] (Figure 1C). The median relative abundance of each group is shown in Figure 1D. In the DILI group, the proportion of primary bile acids increased more than in the HC groups. The relative abundance of all samples is shown in Supplementary Figure S1.

**Figure 2:**
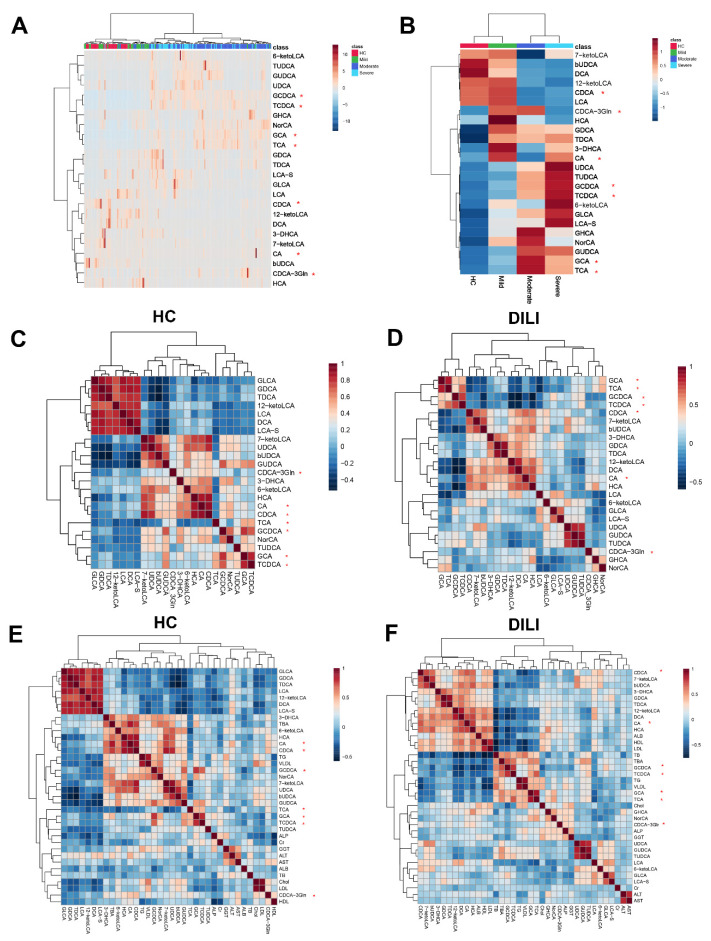
Clustering analysis of bile acid among patients and groups. Bile acids clustering for each sample and each group are shown in (**A**,**B**). Correlation analysis between different bile acids in HC and DILI group were shown in (**C**,**D**). (**E**,**F**) are the correlation analysis between bile acids and biochemical index in HC and DILI group. *: primary bile acid.

Clustering analysis of different samples revealed that the 24 bile acids enabled the discrimination of patients in different groups (Figure 2A). Similarly, clustering analysis of different groups revealed a clear distinction with respect to bile acid concentration (Figure 2B).

The relationship between different bile acids, as well as the relationship between bile acids and liver function-related biochemistry, in the HC group and DILI group was also explored, as shown in Figure 2C–F, and the correlation of bile acids and biochemical indicators in mild, moderate, and severe groups were also drawn in Supplementary Figures S2 and S3. In the HC group, primary and secondary BAs clustered separately better than the DILI group (Figure 2C,D). In the DILI groups, the positive correlation among liver injury-related indicators (ALT, AST) and several BAs (LCA-S, GHCS and NorCA) was observed in the mild group, but was not obvious in the moderate and severe groups.

### 2.3. Differential Bile Acid Analysis of DILI Patients with Different Severity

**Figure 3:**
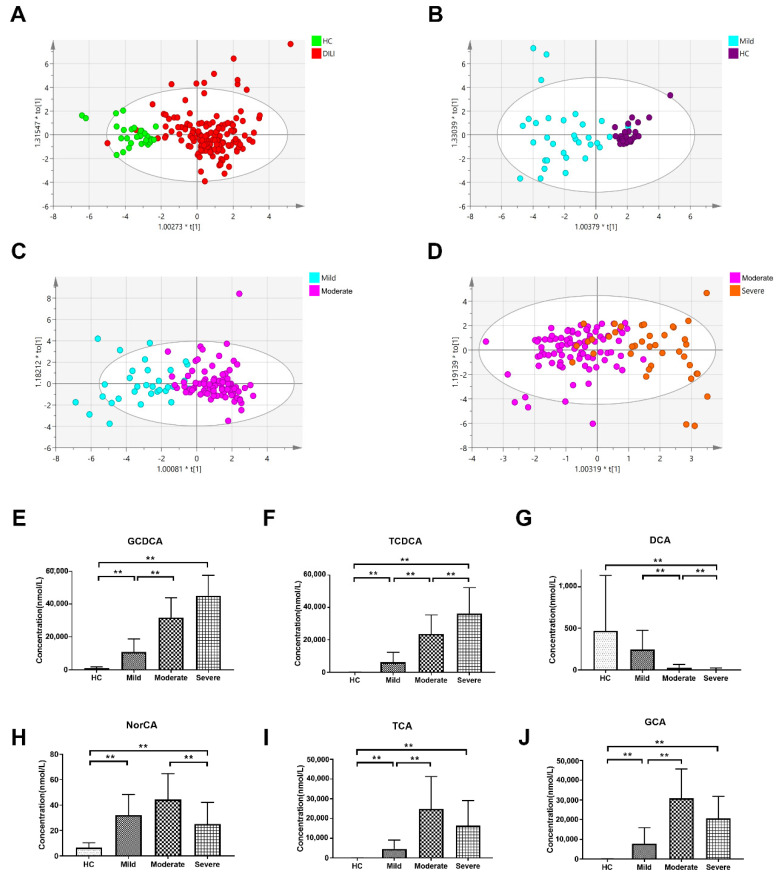
OPLS-DA models of different groups. OPLS-DA models between (**A**) The HC and DILI groups (R2X (cum) = 0.41, R2Y (cum) = 0.442, Q2 (cum) = 0.393), (**B**) HC and mild groups (R2X (cum) = 0.52, R2Y (cum) = 0.719, Q2 (cum) = 0.566), (**C**) mild and moderate groups (R2X (cum) = 0.38, R2Y (cum) = 0.678, Q2 (cum) = 0.518), and (**D**) moderate and severe groups (R2X (cum) = 0.317, R2Y (cum) = 0.541, Q2 (cum) = 0.4). The concentrations of (**E**) GCDCA, (**F**) TCDCA, (**G**) DCA, (**H**) NorCA, (**I**) TCA and (**J**) GCA in different groups. ** *p* < 0.01 and FC > 2. HC: healthy controls.

The OPLS-DA scatter plot clearly discriminated between the HC and DILI groups, and between HC and mild, mild and moderate, as well as moderate and severe groups (Figure 3).

Bile acids with a VIP > 1 and fold change (FC) > 2 (*p* < 0.05) between two groups were selected and are listed in Table 2. A volcano plot between each of the two groups is shown in Supplementary Figure S4. In the DILI group, five bile acids increased (GCA, GCDCA, NorCA, TCA, and TCDCA) and three bile acids (12-ketoLCA, bUDCA, and DCA) decreased in concentration compared to the HC group. In the moderate group, four bile acids increased (GCA, GCDCA, TCA, TCDCA) and five decreased (12-ketoLCA, 7-ketoLCA, CDCA, DCA, and LCA) in quantity. Compared to the moderate group, two bile acids were elevated (TCDCA and UDCA) and three bile acids were reduced (CDCA-3Gln, DCA, and NorCA). Among four groups, there were six bile acids (GCDCD, TCDCA, DCA, NorCA, GCA, and TCA) that showed significant concentration differences at least between two groups. Notably, the changes in the concentration of some bile acids were not consistent with the severity of disease. The concentration levels of TCA, GCA, and NorCA increased in the mild and moderate groups but decreased in the severe group.

### 2.4. Bile Acids for Predicting Severe DILI Patients

**Figure 4:**
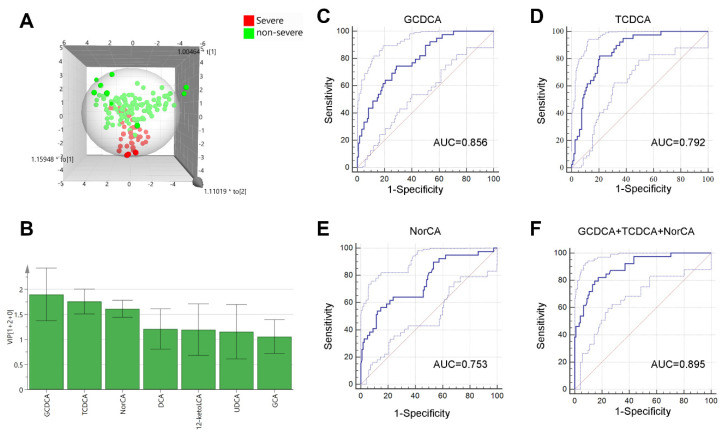
OPLS−DA models and ROC curves of selected bile acids. (**A**) OPLS−DA 3D model between severe and non-severe groups. (**B**) Bile acids with VIP values > 1. ROC curves of GCDCA (**C**), TCDCA (**D**), NorCA (**E**) and the combination of all three bile acids (**F**). The blue line represents the ROC curve and the light blue solid line represents the 95% CI. OPLS-DA: orthogonal partial least squares-discriminant analysis.

To select bile acids that could predict the worsening of DILI patients, we divided all patients into a non-severe group (mild and moderate groups) and a severe group. The OPLS-DA model showed a clear discrimination (Figure 4A). Seven bile acids with VIP > 1 were selected for further analysis (Figure 4B).

Meanwhile, univariate and multivariant logistic analyses were performed (Table 3). Univariate logistic analysis revealed that 10 bile acids were related to the severity. Among them, 6 bile acids (GCDCA, TCDCA, NorCA, DCA, 12-ketoLCA and UDCA) with VIP >1 were selected for multivariate analysis. It revealed that NorCA, GCDCA, and TCDCA were independent risk factors for differentiating severe DILI patients. The area under the receiver operating characteristic (AUC) of GCDCA, TCDCA, and NorCA were 0.856, 0.792, and 0.753, respectively. Together, these three bile acids had an AUROC of 0.895 for predicting the severity of DILI patients. In addition, we calculated the AUCs of GCDCA, TCDCA and NorCA as a combined form, and the AUCs and comparison of these models for differentiating severe DILI patients was listed in Supplementary Table S5.

## 3. Discussion

Metabolomics has been increasingly used to study liver diseases such as DILI, to help reveal the pathophysiological processes and identify specific biomarkers for diagnosing and predicting prognoses [11,12]. Soga et al. [13] analyzed low-molecular-weight metabolites from patients with nine types of liver disease and healthy controls. They found that γ-glutamyl dipeptides could be used to distinguish among different liver diseases. This result indicates that certain metabolites could have potential for discriminating between different liver diseases. In another study [14], metabolomic analysis was used to discriminate between different DILI phenotypes. That study revealed that metabolites could complement the concise information drawn out by the ALT and ALP DILI classification. Furthermore, in idiosyncratic DILI caused by *Polygonum multiflorum* [15], metabolomics analysis showed that the 25 metabolites produced from glycerophospholipid metabolism, sphingolipid metabolism, fatty acid metabolism, and so forth, could clearly distinguish between susceptible and tolerant groups.

Compared to untargeted metabolomics, metabolite detection using targeted metabolomics can quantify results and produce more accurate results. Untargeted metabolomic compound identification has been poor and leaves an overwhelming number of unidentified peaks [16]. Moreover, it cannot accurately quantify the concentration of metabolites. Jacek Klepacki et al. [17] found that whenever assessing a specific pathway such as amino acids is the focus of interest, a targeted metabolomics assay seems preferable to a non-targeted metabolomics assay. However, targeted metabolomics cannot explore the comprehensive characteristics of all metabolic ion peaks in the sample, so it is impossible to realize the macroscopic detection and analysis of metabolomics. Therefore, a research strategy that combines untargeted metabolomics with targeted metabolomics could help analyze metabolite changes more comprehensively and accurately.

In a previous study [10], we found that primary bile acid biosynthesis and alpha-linolenic acid metabolism pathways were related to the severity of DILI. Meanwhile, several cytokines were distinct between patients with severe and non-severe DILI, which indicated that the metabolites related to these pathways might be related to the immune responses in patients. Bile acids, synthesized from cholesterol exclusively in hepatocytes, are known as amphipathic molecules. These facilitate hepatobiliary secretion of lipids, endogenous metabolites, and xenobiotics. Current evidence supports that bile acids can result in necrotic liver cell death, and the factors released by necrotic cell death may work as damage-associated molecular patterns and initiate a sterile inflammatory response [18]. Generally, bile acid receptors include nuclear receptors and G protein-coupled receptors, but Zhuang et al. [19] discovered more than 600 BA-interacting protein targets using SILAC-based quantitative proteomic technologies. This expanded the current understanding of BA-mediated pathways in human metabolism and disease.

Our targeted metabolomics analysis revealed that GCDCA, TCDCA, and NorCA were related to the severity of DILI. GCDCA was reported to induce apoptosis in hepatocytes [20,21] and induce stemness and chemoresistance in hepatocellular carcinoma cells [22]. It induced endoplasmic reticulum (ER) stress-mediated calcium release, and increased activities of calpain and caspase-12, which triggered apoptosis. TCDCA was reported to activate a phosphatidylinositol 3-kinase (PI3-K)-mediated survival pathway, which induced liver damage and hepatocyte apoptosis [23]. NorCA, also known as norcholate, belongs to the class of organic compounds known as trihydroxy bile acids, alcohols, and derivatives, and so far, very few articles have been published on NorCA. It is biotransformed by intestinal bacterium and appeared in the bile predominantly as the unconjugated form [24]. NorCA was detected in patients with a rare inherited disease called cerebrotendinous xanthomatosis [25,26], which is characterized biochemically by storage of cholestanol in most tissues, decreased synthesis of bile acids, and accumulation of large amounts of bile alcohols. In 1977, Alme et al. [27] detected and quantified NorCA in the urine of healthy controls and patients with PBC or congenital intrahepatic cholestasis. In 1982, Amuro et al. [28] found that NorCA was the major trhydroxy bile acid which was found in the urine of patients with liver cirrhosis. These results indicate that NorCA might increase in patients with liver function abnormality. Recently, Tian et al. [29] found that NorCA is one of the most significantly changed 6 bile acids in DILI patients, which is consistent with our results. However, the authors did not discuss the meaning of the changes in this paper, and the underlying mechanism is not clear. In DILI, hepatotoxic substances damage liver cells and disturb bile acid metabolism. As a result, the accumulated bile acids might further aggravate liver damage and delay the recovery of liver cells. In our results, GCDCA + TCDCA + NorCA owns the maximum AUC value for differentiating severe DILI, however it does not show significance when compared with GCDCA, GCDCA + NorCA and GCDCA + TCDCA. A multicenter large sample cohort study is needed to verify its validity and specificity.

An animal experiment showed that decreasing plasma BA concentration by Sirtuin1 can alleviate cholestatic liver injury in mice [30]. In another animal study [31], inhibition of the apical sodium-dependent bile salt transporter (ASBT/SLC10A2) on the ileal brush border membrane reduced biliary BA concentrations in Mdr2−/− mice, which attenuated cholestatic liver and bile duct injury. For DILI patients, the accumulation of certain bile acids possibly aggravated the liver cells and exacerbated local inflammatory reactions, thereby further aggravating the liver injury.

Interestingly, compared to the moderate group, the TBA concentration did not increase significantly in the severe group and the difference was only manifested as changes in the content of different types of bile acids. Meanwhile, ALP and GGT were lower in the severe group than in the moderate group, both of which are related to cholestasis. These changes suggest that there may be some characteristic modifications in bile acid-related pathways during disease exacerbation. Xie et al. [32] found that bile acid reabsorption increased intestinal luminal pH and facilitated the conversion of intestinal ammonium to ammonia, leading to abnormally high levels of neurotoxic ammonia and cytotoxic BAs in the blood and brain. This exacerbated hepatic encephalopathy and liver failure. These results indicate that bile acids can also influence other metabolic pathways, which in turn aggravate the disease. In addition, bile acids could affect the disease process through modulation of immune responses. Biagioli et al. [33] found that deletion of Gpbar1, a G protein–coupled receptor for secondary bile acids, worsened the severity of APAP-induced liver injury. The CCL2/CCR2 axis at the sinusoidal cell/macrophage interface was modulated by Gpbar1, providing a novel target for the treatment of liver damage caused by APAP.

This study had several limitations. Firstly, it is still uncertain whether the characteristics of bile acid changes found in this study are specific to DILI patients. However, they would be helpful to distinguish non-severe from severe DILI. Secondly, the association between mortality and these characteristics was not analyzed due to the small number of deaths. A multicenter study cohort with more patients is necessary to verify the findings of this study. In addition, the relationship between changes in bile acids and the inflammatory response in DILI patients’ needs further experimental exploration, and the changes in bile acid-related pathways and in upstream and downstream pathway molecules need to be further studied.

## 4. Materials and Methods

### 4.1. Patients

Patients diagnosed with idiosyncratic DILI were enrolled from 1 August 2015 to 30 June 2018. Intrinsic DILI were excluded due to the different pathophysiological mechanisms. IgM antibodies specific for Hepatitis A, B, C, D, and E, as well as cytomegalovirus (CMV) and Epstein–Barr virus (EBV) were measured to exclude viral hepatitis. Anti-nuclear antibody and antineutrophil cytoplasmic antibodies were also measured to rule out autoimmune hepatitis. The patients’ clinical data were obtained from the electronic medical record system.

Serum samples from patients without DILI were collected from the health examine center and used as health controls (HCs).

This study was conducted in compliance with the ethical principles of the Declaration of Helsinki and was approved by the Ethics Committee of the First Affiliated Hospital, Zhejiang University School of Medicine. The enrolled patients or their legal representatives provided written informed consent.

### 4.2. Criteria

Patients hospitalized in the First Affiliated Hospital of Zhejiang University, between January 2016 and October 2019, with a medication history and a hepatic biochemical abnormality were studied. The patients who met one or more of the following criteria were enrolled: aspartate aminotransferase (AST) or alanine aminotransferase (ALT) > 5X the upper limit of normal (ULN); serum levels of total bilirubin (TB) > 2.5 mg/dL and elevated AST, ALT, or ALP; and international normalized ratio (INR) > 1.5 with elevated AST, ALT, or ALP.

The exclusion criteria included liver injury caused by APAP or hepatitis viruses; metabolic liver diseases, autoimmune liver diseases, or liver cancer; also, patients with a liver or bone-marrow transplant prior to enrollment were excluded.

The R value was defined as the ratio of serum levels of ALT (as a multiple of its ULN) to that of ALP (as a multiple of its ULN). In the clinical characterization of DILI, the Hepatocellular DILI was defined as R ≥ 5, cholestatic DILI as R ≤ 2, and mixed DILI as 2 < R < 5 [34]. The Roussel Uclaf Causality Assessment Method (RUCAM) was used to evaluate the causality of any relationships identified [35]. The causal relationship between the agent and the liver-injury event was categorized as highly probable (>8), probable (6–8), possible (3–5), unlikely (1 or 2), or excluded (0). The severity was graded according to the International DILI Expert Working Group [36], as shown in Supplementary Table S6.

### 4.3. Targeted Metabolomic Analysis

#### 4.3.1. Sample Preparation

Blood samples were obtained on the day following hospitalization. The serum was separated by centrifugation at 3500 rpm for 10 min and was stored at −80 °C. The thawed serum (20 μL) was mixed with a 180 µL acetonitrile/methanol (*v/v* = 8:2) mixed solvent containing 10 µL, an internal standard. After shaking and centrifugation, the supernatant was transferred to a 600 μL centrifuge tube and then freeze-dried. The dried actual sample, standard curve, and quality control sample powder were reconstituted with 1:1 acetonitrile/methanol (80/20) and an ultrapure water-mixed solvent. After they completely dissolved, the supernatant was transferred to a 96-well plate for analysis.

#### 4.3.2. On-Board Testing

BA profiling and quantitation were performed using published methods with modifications [37]. Serum BAs were assayed using an UPLC-MS/MS system (ACQUITY UPLCXevo TQ-S, Waters Corp., Milford, MA, USA). The solvent and equipment preparation were as described previously [38]. A total of 24 types of bile acid (detailed in Supplementary Table S3) were targeted for detection. Bile acid standards were bought from Steraloids, Inc. (Newport, RI, USA) and TRC Chemicals (Toronto, ON, Canada). Specific bile acid chromatograms, as well as qualitative and quantitative ion mass and isotope internal standard parameters, were detailed in a previous study [37]. The quality control samples were prepared with the test samples and injected at every 14 test samples throughout the process.

### 4.4. Statistic Analysis

The raw data exported by UPLC-MS/MS were processed using QuanMET software (v1.0, Metabo-Profile, Shanghai, China). The concentration and peak area of standards were used to build a standard curve and calculate the sample concentration. The calculated concentrations of bile acids in all samples were imported into SIMCA-P+ software (v. 14.1, Umetrics, Sweden) for multivariate analysis, including principal component analysis (PCA) and orthogonal partial least squares-discriminant analysis (OPLS-DA). An independent sample non-parametric test judgment was used to test the significant differences between the groups (*p* < 0.05) and the variables of importance (VIP) values in the OPLS-DA model were used to identify potential biomarkers.

Statistical analyses were performed using Statistical Package for the Social Sciences (SPSS, v.22.0; SPSS, Inc., Chicago, IL, USA) software. GraphPad Prism (v 7.0, San Diego, California, CA, USA) was used for graphing. Continuous data are expressed as means ± standard deviations or medians with interquartile ranges (p25, p75), while categorical data are expressed as numbers (percentages). The Student’s *t*-test was used to compare continuous data for normal distributions and the Mann–Whitney U-test was used to compare non-parametric data. Categorical data and ordered categorical data were compared using chi-square tests. The area under the receiver operating characteristic curve (AUROC) of the various prognostic scoring systems was compared by Z-tests using Delong’s method.

## 5. Conclusions

Idiosyncratic DILI patients with different disease severity have specific bile acid metabolomic characteristics. NorCA, GCDCA, and TCDCA are independent risk factors for differentiating severe idiosyncratic DILI patients from more mild patients. These markers may have the potential to predict the severity of DILI.

## Figures and Tables

**Table 1:** Clinical characteristics of different severity groups of DILI patients.

	Health Control (*n* = 31)	Grade 1 (Mild) *n* = 32	Grade 2 (Moderate) *n* = 90	Grade 3 (Severe) *n* = 39	*p* Value
Age, years	50.1 ± 14.8	51.4 ± 14.9	51.1 ± 13.8	52.1 ± 15.5	0.937
Female%	16(51.6)	26(81.3)	55(61.1)	29(74.4)	0.071
Alcohol use	7(22.6)	5(15.6)	17(18.9)	6(15.4)	0.852
Hypertension	7(22.6)	7(21.9)	20(22.2)	6(15.4)	0.661
Latency					
<5 days	/	6(18.8)	7(7.8)	1(2.6)	0.179
5 days–90 days		23(71.9)	76(84.4)	34(87.2)	
>90 days		3(9.4)	7(7.8)	4(10.3)	
Liver biochemistries					
WBC (10^9^/L)	6.6(5.4, 7.6)	4.6(3.8, 5.3)	5(4.3, 6.8)	7.2(4.7, 9.3) ##	<0.001
ALB (g/L)	47.5(45.1, 48.8)	40.6(38, 42.6)	36.4(32.5, 39.1) ***	31.4(28.2, 33.5) ###	<0.001
ALT (U/L)	14(12, 22)	401(246, 655.8)	360.5(136.8, 715.8)	337(131, 617)	0.448
AST (U/L)	19(17, 21.5)	200.5(96.3, 318.3)	248(88.8, 474.3)	211(117, 336)	0.65
TBA (μmol/L)	5(3, 6)	13.6(7, 20.6)	151.4(117.9, 194.5) ***	177(137, 210)	<0.001
ALP (U/L)	66(55, 82)	120.5(98.3, 147.3)	147.5(113.5, 217.8)	116(99, 134) ###	<0.001
GGT (U/L)	24(14, 35)	124(70, 194.8)	128(73.5, 305) **	72(41, 122) ###	<0.001
TB (μmol/L)	12(8, 16)	13.3(9.7, 18.6)	241.3(111.6, 358.6) ***	291(240.2, 443)	<0.001
TG (mmol/L)	1.7(0.97, 2.31)	1.15(0.74, 1.49)	2.54(1.78, 3.52) ***	1.63(1.06, 2.4) ###	<0.001
Cholesterol (mmol/L)	4.54(4.06, 5.08)	3.41(3.18, 4.05)	3.52(2.81, 4.61)	2.71(2.25, 3.19) ###	<0.001
HDL (mmol/L)	1.13(0.97, 1.31)	1.07(0.79, 1.24)	0.19(0.13, 0.43) ***	0.15(0.12, 0.33)	<0.001
LDL (mmol/L)	2.34(2.08, 3.01)	1.78(1.37, 2.22)	0.67(0.23, 1.42) ***	0.68(0.18, 1.37)	<0.001
VLDL (mmol/L)	0.88(0.65, 1.13)	0.69(0.55, 0.91)	2.26(1.44, 3.45) ***	1.45(0.6, 2.26) ###	<0.001
INR	/	0.99(0.96, 1.05)	1.08(0.97, 1.23) *	1.62(1.53, 2.16) ###	<0.001
Pattern of liver injury (%)					
Hepatocellular	/	26(81.3)	57(63.3)	25(64.1)	0.025
Cholestatic		6(18.8)	12(13.3)	9(23.1)	
Mixed		0(0)	21(23.3)	5(12.8)	
RUCAM score					
Highly probable (>8)	/	3(9.4)	1(1.1)	1(2.6)	0.094
Probable (6–8)		21(65.6)	58(64.4)	30(76.9)	
Possible (3–5)		8(25)	31(34.4)	8(20.5)	

Abbreviations: WBC: white blood cell; ALB: albumin; ALT: alanine aminotransferase; AST: aspartate aminotransferase; TBA: total bile acid; ALP: phosphatase alkaline; GGT: gamma-glutamyl transpeptidase; TB: total bilirubin; TG: Triglyceride; HDL: high-density lipoprotein; LDL: low-density lipoprotein; VLDL: very low-density lipoprotein; INR: international normalized ratio; RUCAM: Roussel Uclaf Causality Assessment Method. *: *p* < 0.05 moderate group vs. mild group; **: *p* < 0.01 moderate group vs. mild group; ***: *p* < 0.001 moderate group vs. mild group. ##: *p* < 0.01 severe group vs. moderate group; ###: *p* < 0.001 severe group vs. moderate group.

**Table 2:** Differential bile acids between different groups.

Bile Acid	Type	VIP	FC	log2(FC)	Differentially Expression	*p* Value
DILI vs. HC						
12-ketoLCA	Secondary	1.109	0.359	−1.476	↓	7.28 × 10^−5^
bUDCA	Secondary	1.043	0.185	−2.436	↓	4.61 × 10^−8^
DCA	Secondary	1.301	0.139	−2.846	↓	3.28 × 10^−11^
GCA	Primary	1.501	147.95	7.209	↑	1.05 × 10^−14^
GCDCA	Primary	1.551	34.106	5.092	↑	4.94 × 10^−20^
NorCA	Secondary	1.4	5.645	2.497	↑	2.47 × 10^−14^
TCA	Primary	1.353	926.55	9.856	↑	5.05 × 10^−10^
TCDCA	Primary	1.446	618.02	9.272	↑	3.21 × 10^−14^
Mild vs. HC						
6-ketoLCA	Secondary	1.026	3.526	1.818	↑	7.68 × 10^−5^
GCA	Primary	1.218	48.529	5.601	↑	2.36 × 10^−6^
GCDCA	Primary	1.408	11.881	3.571	↑	4.05 × 10^−9^
GDCA	Secondary	1.215	4.964	2.312	↑	8.28 × 10^−7^
GHCA	Secondary	1.294	4544	12.15	↑	1.66 × 10^−7^
GLCA	Secondary	1.015	2.865	1.518	↑	5.03 × 10^−3^
GUDCA	Secondary	1.135	45.726	5.515	↑	3.20 × 10^−4^
HCA	Secondary	1.017	5.565	2.476	↑	5.91 × 10^−5^
NorCA	Secondary	1.402	4.88	2.287	↑	5.27 × 10^−12^
TCA	Primary	1.284	226.42	7.823	↑	4.68 × 10^−7^
TCDCA	Primary	1.33	163.05	7.349	↑	6.90 × 10^−7^
TDCA	Secondary	1.325	18.522	4.211	↑	7.58 × 10^−8^
TUDCA	Secondary	1.162	363.28	8.505	↑	4.18 × 10^−4^
Moderate vs. Mild						
12-ketoLCA	Secondary	1.217	0.223	−2.164	↓	6.47 × 10^−8^
7-ketoLCA	Secondary	1.239	0.221	−2.18	↓	4.48 × 10^−9^
CDCA	Secondary	1.081	0.186	−2.423	↓	1.90 × 10^−6^
DCA	Secondary	1.471	0.108	−3.212	↓	2.79 × 10^−14^
GCA	Primary	1.395	3.955	1.984	↑	1.40 × 10^−13^
GCDCA	Primary	1.49	2.947	1.559	↑	2.37 × 10^−15^
LCA	Secondary	1.084	0.355	−1.492	↓	7.76 × 10^−4^
TCA	Primary	1.351	5.406	2.435	↑	3.24 × 10^−10^
TCDCA	Primary	1.428	3.842	1.942	↑	1.00 × 10^−12^
Severe vs. Moderate						
CDCA-3Gln	Primary	1.136	0.433	−1.207	↓	1.16 × 10^−4^
DCA	Secondary	1.177	0.368	−1.442	↓	0.010523
NorCA	Secondary	1.692	0.565	−0.824	↓	6.78 × 10^−7^
TCDCA	Primary	1.398	1.537	0.62	↑	1.59 × 10^−6^
UDCA	Secondary	1.037	1.808	0.854	↑	0.014075

Abbreviations: 12-ketoLCA: 12-ketolithocholic acid; 6-ketoLCA: 6-ketolithocholic acid; 7-ketoLCA: 7-ketolithocholic acid; CDCA: chenodeoxycholic acid; CDCA-3Gln: Chenodeoxycholic acid-3-β-D-glucuronide; DCA: deoxycholic acid; GCA: glycocholic acid; GCDCA: glycochenodeoxycholate; GDCA: glycodeoxycholic acid; GHCA: glycohyocholate; GLCA: glycolithocholate; GUDCA: glycoursodeoxycholic acid; HCA: hyocholic acid; LCA: lithocholic acid; NorCA: Nor Cholic acid; TCA: taurocholic acid; TCDCA: taurochenodeoxycholate; TDCA: taurodeoxycholate; TUDCA: tauroursodeoxycholic acid; UDCA: ursodeoxycholic acid. ↑: increased in the front group when compared with the back group, ↓: decreased in the front group when compared with the back group.

**Table 3:** Univariate and multivariate analysis of bile acids for differentiating severe DILI.

Variable	Univariate Analysis		Multivariate Analysis	
	OR (95% CI)	*p* Value	OR (95% CI)	*p* Value
12-ketoLCA (nmol/L)	0.665(0.486, 0.908)	0.01		
HCA (nmol/L)	0.847(0.775, 0.925)	<0.001		
NorCA (nmol/L)	0.945(0.919, 0.972)	<0.001	0.941(0.912, 0.972)	<0.001
DCA (nmol/L)	0.965(0.943, 0.987)	0.002		
bUDCA (nmol/L)	0.981(0.967, 0.995)	0.008		
CDCA-3Gln (nmol/L)	0.995(0.992, 0.998)	<0.001		
6-ketoLCA (nmol/L)	1.042(1.003, 1.082)	0.034		
TCDCA (umol/L)	1.084(1.051, 1.117)	<0.001	1.061(1.016, 1.107)	0.007
GCDCA (umol/L)	1.111(1.067, 1.156)	<0.001	1.064(1.020, 1.110)	<0.001
UDCA (umol/L)	1.365(1.121, 1.663)	0.002		

Abbreviations: 12-ketoLCA: 12-ketolithocholic acid; 6-ketoLCA: 6-ketolithocholic acid; bUDCA: 3β-Ursodeoxycholic Acid; CDCA-3Gln: Chenodeoxycholic acid-3-β-D-glucuronide; DCA: deoxycholic acid; GCDCA: glycochenodeoxycholate; HCA: hyocholic acid; NorCA: Nor Cholic acid; TCDCA: taurochenodeoxycholate; UDCA: ursodeoxycholic acid.

## Data Availability

The data presented in this study are available on request from the corresponding author. The data are not publicly available due to protection of the privacy of patients and the innovation and uniqueness of following researches using these data.

## Supplementary Materials

The following are available online at https://www.mdpi.com/article/10.3390/metabo11120852/s1, Figure S1: relative abundance of primary and secondary bile acids in all samples, Figure S2: Correlation of different bile acids in different groups, Figure S3: Correlation of different bile acids and biochemical indexes in different groups, Figure S4: volcano plots between each of the two groups, Table S1: Characteristics of healthy controls and drug-induced liver injury patients, Table S2: Suspected drugs causing DILI, Table S3: Full name and type of detected bile acids, Table S4: Concentration levels of 24 bile acids in different groups, Table S5: AUC values and comparison of GCDCA, TCDCA and NorCA as a single factor or combined form for predicting the severity of DILI, Table S6: Severity grading criteria of International DILI Expert Working Group.
