# Supplementary material for: Targeted Metabolomics Analysis of Bile Acids in Patients with Idiosyncratic Drug-Induced Liver Injury
Source: Metabolites. 2021 Dec 8;11(12):852. doi: 10.3390/metabo11120852 (PMC8706581; doi:10.3390/metabo11120852)
Supplement: Supplementary file 1 [file metabolites-11-00852-s001.zip › SU Tables-revised.pdf]

Supplementary table S1 Characteristics of healthy controls and drug-induced liver injury patients

	Health Control (n=31)	DILI group (n=161)	P value
Age, years	50.1±14.8	51.4±14.4	0.649
Female%	16(51.6)	110(68.3)	0.073
Liver biochemistries			
WBC (10^9/L)	6.6（5.4,7.6)	5.2（4.3,7.3)	0.015
RBC (10^9/L)	4.7（4.3,5.2)	4.1（3.8,4.5)	<0.001
PLT (10^9/L)	223（184,267)	189（140.5,249.5)	0.013
ALB (g/L)	47.5（45.1,48.8)	35.8（32,39.5)	<0.001
ALT (U/L)	14（12,22)	355（142,667)	<0.001
AST (U/L)	19（17,21.5)	226（93.5,371.5)	<0.001
TBA (μmol/L)	5（3,6)	145.7（45.5,187)	<0.001
ALP (U/L)	66（55,82)	129（106,177.5)	<0.001
GGT (U/L)	24（14,35)	110（56.5,201)	<0.001
TB (μmol/L)	12（8,16)	214（51.8,343.4)	<0.001
TG (mmol/L)	1.7（0.97,2.31)	2.05（1.23,3.2)	0.049
Cholesterol (mmol/L)	4.54（4.06,5.08)	3.3（2.71,4.19)	<0.001
HDL (mmol/L)	1.13（0.97,1.31)	0.25（0.13,0.7)	<0.001
LDL (mmol/L)	2.34（2.08,3.01)	1.06（0.28,1.66)	<0.001
VLDL (mmol/L)	0.88（0.65,1.13)	1.66（0.77,2.77)	<0.001

Abbreviations: WBC: white blood cell; ALB: albumin; ALT: alanine aminotransferase; AST: aspartate aminotransferase; TBA: total bile acid; ALP: phosphatase alkaline; GGT: gamma－glutamyl transpeptidase; TB: total bilirubin; TG: Triglyceride; HDL: high-density lipoprotein; LDL: low-density lipoprotein; VLDL: very low-density lipoprotein.

Supplementary table S2 Suspected drugs causing DILI

	Grade 1(mild) n=32	Grade 2(moderate) n=90	Grade 3(severe) n=39
TCM or HDS	21(65.6)	78(86.7)	25(64.1)
Polygonum Multiflorum	4(12.5)	8(8.9)	2(5.1)
Fructus Psoraleae	1(3.1)	3(3.3)	3(7.7)
no TCM or HDS	11(34.4)	12(13.3)	14(35.9)
Anti-Tuberculosis	1(3.1)	2(4)	4(10.3)
cephalosporin	1(3.1)	1(1.1)	0(0)
Azithromycin	1(3.1)	0(0)	0(0)
Amoxicillin	0(0)	1(1.1)	0(0)

Supplementary Table S3 Full name and type of detected bile acids

Short name	Full name	type
12-ketoLCA	12-ketolithocholic acid	Secondary BA
3-DHCA	3-dehydrocholic acid	Secondary BA
6-ketoLCA	6-ketolithocholic acid	Secondary BA
7-ketoLCA	7-ketolithocholic acid	Secondary BA
bUDCA	3β-Ursodeoxycholic Acid	Secondary BA
CA	cholic acid	Primary BA
CDCA	chenodeoxycholic acid	Primary BA
CDCA-3Gln	Chenodeoxycholic acid-3-β-D-glucuronide	Primary BA
DCA	deoxycholic acid	Secondary BA
GCA	glycocholic acid	Primary BA
GCDCA	glycochenodeoxycholate	Primary BA
GDCA	glycodeoxycholic acid	Secondary BA
GHCA	glycohyocholate	Secondary BA
GLCA	glycolithocholate	Secondary BA
GUDCA	glycoursodeoxycholic acid	Secondary BA
HCA	hyocholic acid	Secondary BA
LCA	lithocholic acid	Secondary BA
LCA_S	lithocholic acid 3 sulfate	Secondary BA
NorCA	Nor Cholic acid	Secondary BA
TCA	taurocholic acid	Primary BA
TCDCA	taurochenodeoxycholate	Primary BA
TDCA	taurodeoxycholate	Secondary BA
TUDCA	tauroursodeoxycholic acid	Secondary BA
UDCA	ursodeoxycholic acid	Secondary BA

Supplementary table S4 Concentration levels of 24 bile acids in different groups.

	**Health Control n=31**	**Grade 1(mild) n=32**	**Grade 2(moderate) n=90**	**Grade 3(severe) n=39**
12-ketoLCA (nmol/L)	11.2±14.2	11.7±11	2.6±6.1**	1.1±1.4##
3-DHCA (nmol/L)	4.8±3.7	15.5±18.6§§	6.4±6.4**	9.9±22.5
6-ketoLCA (nmol/L)	2.3±1.1	8±7.4§§	5.7±6.1	13.9±28.9
7-ketoLCA (nmol/L)	34±60.3	36±35.8	7.9±13.3**	28.3±93.2
b-UDCA (nmol/L)	435±481.4	283±579.8§	35.7±50.5**	19.2±25.8#
CA (nmol/L)	413.9±651.9	778.6±1010.9§§	355.9±556.6**	726.4±3811.8##
CDCA (nmol/L)	818.5±838.7	882.6±1334.8	164.6±182.4**	242.2±261.5
CDCA-3Gln (nmol/L)	142.4±222.7	318.2±519.4§§	324.8±282.9	140.4±111.2##
DCA (nmol/L)	469.9±665.7	244.4±231.7	26.4±39.3**	9.8±15.1##
GCA（μmol/L）	0.16±0.16	7.8±8.17§§	30.88±14.83**	20.63±11.15##
GCDCA（μmol/L）	0.9±0.9	10.8±7.9§§	31.7±12.1**	44.8±12.5##
GDCA (nmol/L)	189.1±218.6	938.7±729.1§§	751.2±807	587.1±908.3##
GHCA (nmol/L)	0.07±0	318.1±299.6§§	688.1±969.5	357.6±939.9##
GLCA (nmol/L)	14.5±24.8	41.5±45.6§§	53.5±91.5	69.6±59.7##
HCA (nmol/L)	9.1±11.4	50.7±52.4§§	13.6±37.6**	2±5
LCA (nmol/L)	7.7±9	8.2±9.7§§	2.9±6.4**	3.3±5##
LCA_S (nmol/L)	27.5±41.9	44.6±65	45.6±45.3**	61.4±62.8
NorCA (nmol/L)	6.6±3.8	32.2±16.2§§	44.4±20.4	25.3±17.3##
TCA（μmol/L）	0.02±0.03	4.6±4.5§§	24.8±16.4**	16.4±12.7##
TCDCA（μmol/L）	0.038±0.066	6.1±6.1§§	23.5±11.8**	35.1±14.6##
TDCA (nmol/L)	17.3±23.4	321.1±275.7§§	297.1±343.8**	250.7±430.9##
GUDCA（μmolL）	0.1±0.3	5.8±8.3§§	25.6±25.9	24.2±21.4##
TUDCA（μmol/L）	0.003±0.01	1±1.48§§	6.99±9.87**	10.83±19.52
UDCA（μmol/L）	0.19±0.22	0.53±0.96§	1.05±1.5	1.94±2.32

*: p<0.05 moderate group vs mild group; **: p<0.01 moderate group vs mild group;. #: p<0.05 severe group vs moderate group; ##: p<0.01 severe group vs moderate group; §: p<0.05 severe group vs moderate group; §§: p<0.01 severe group vs moderate group.

Supplementary table S5 AUC values and comparison of GCDCA, TCDCA and NorCA as a single factor or combined form for predicting the severity of DILI.

Model	AUC	P value (*v.s.* GCDCA+TCDCA+NorCA)
GCDCA+TCDCA+NorCA	0.895	
TCDCA	0.792	0.007
NorCA	0.753	<0.001
GCDCA	0.856	0.135
TCDCA+NorCA	0.861	0.040
GCDCA+NorCA	0.876	0.196
GCDCA+TCDCA	0.867	0.230

Supplementary Table S6 Severity grading criteria of International DILI Expert Working Group

category	severity	Description
1	mild	ALT ≥5 or ALP ≥2 and TBL <2 ULN
2	moderate	ALT ≥5 or ALP ≥2 and TBL ≥2 ULN, or symptomatic hepatitis
3	severe	ALT ≥5 or ALP ≥2 and TBL ≥2 ULN, or symptomatic hepatitis and 1 of the following criteria:<br>- INR ≥1.5<br>- Ascites and/or encephalopathy, disease duration <26 weeks, and absence of underlying cirrhosis<br>- Other organ failure due to DILI
4	fatal/transplantation	Death or liver transplantation due to DILI
