# Supplementary figures and images for: Targeted Metabolomics Analysis of Bile Acids in Patients with Idiosyncratic Drug-Induced Liver Injury
Source: Metabolites. 2021 Dec 8;11(12):852. doi: 10.3390/metabo11120852 (PMC8706581; doi:10.3390/metabo11120852)

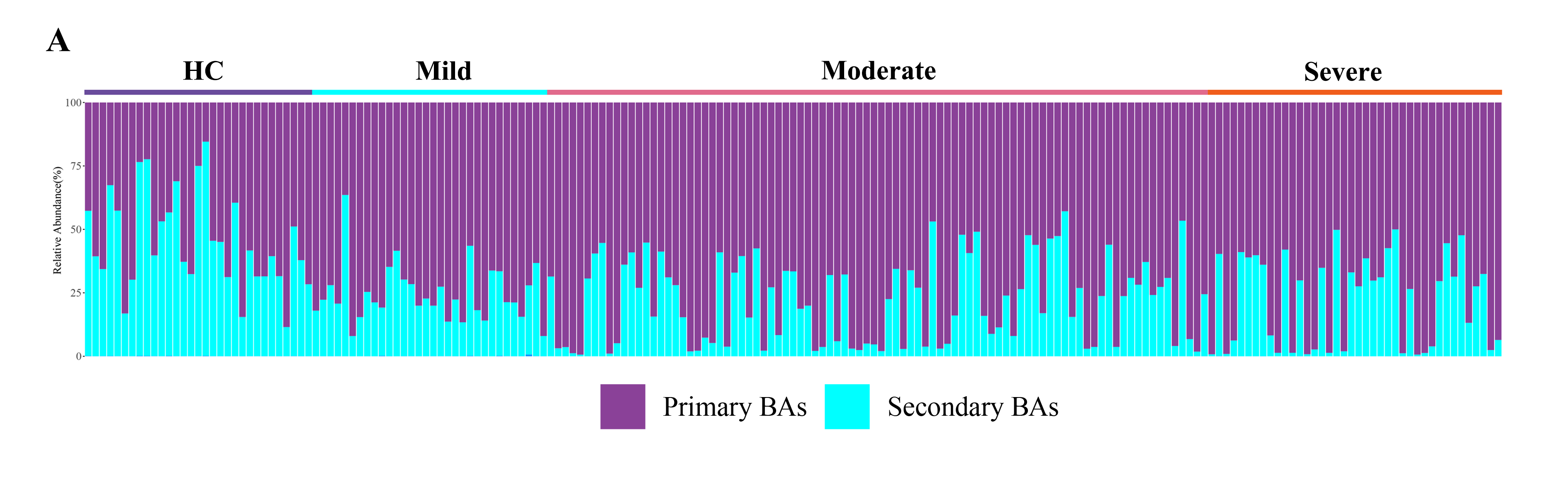

Supplement: Supplementary file 1 [file metabolites-11-00852-s001.zip › Figure S1.tif]

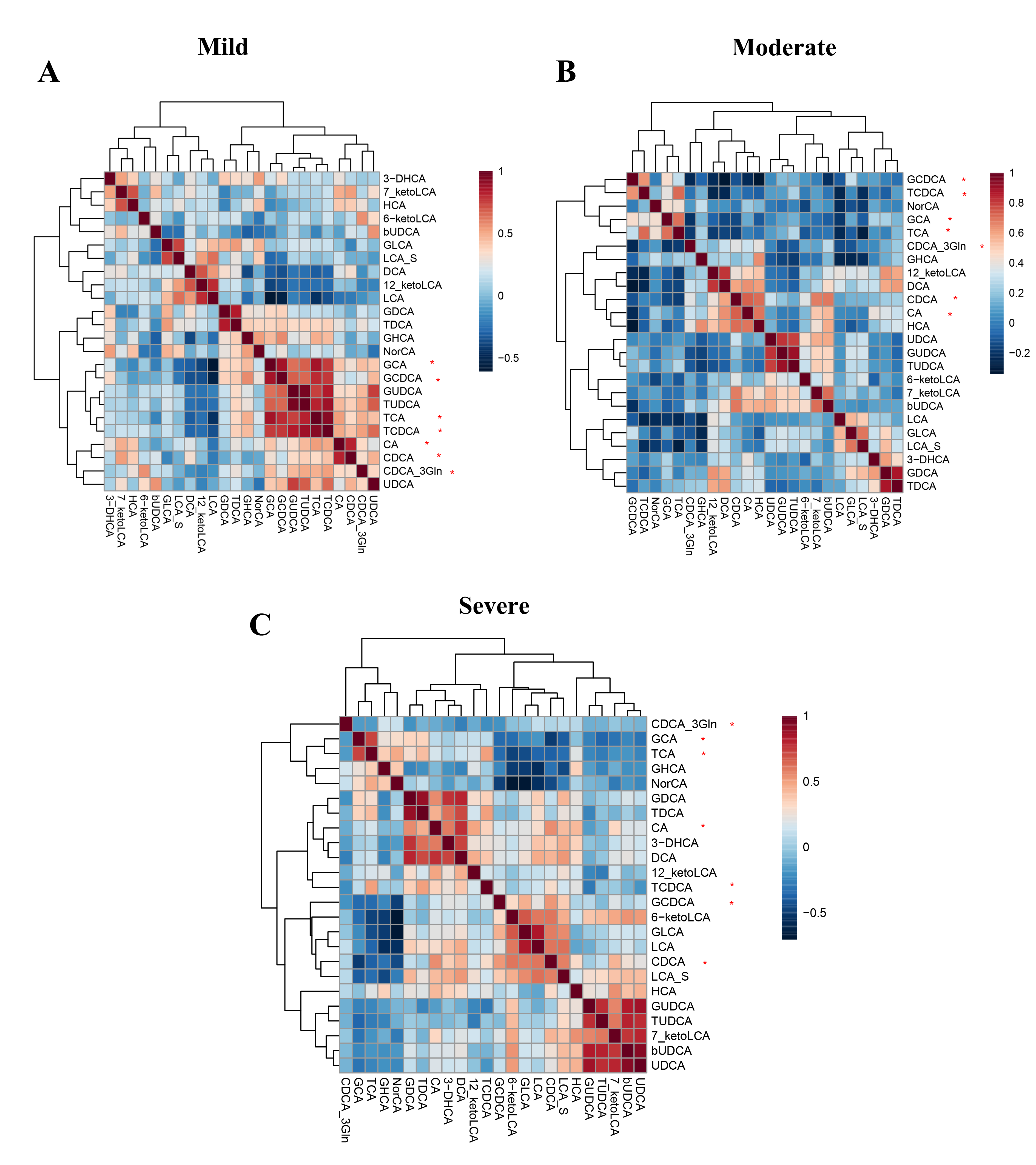

Supplement: Supplementary file 1 [file metabolites-11-00852-s001.zip › Figure S2.tif]

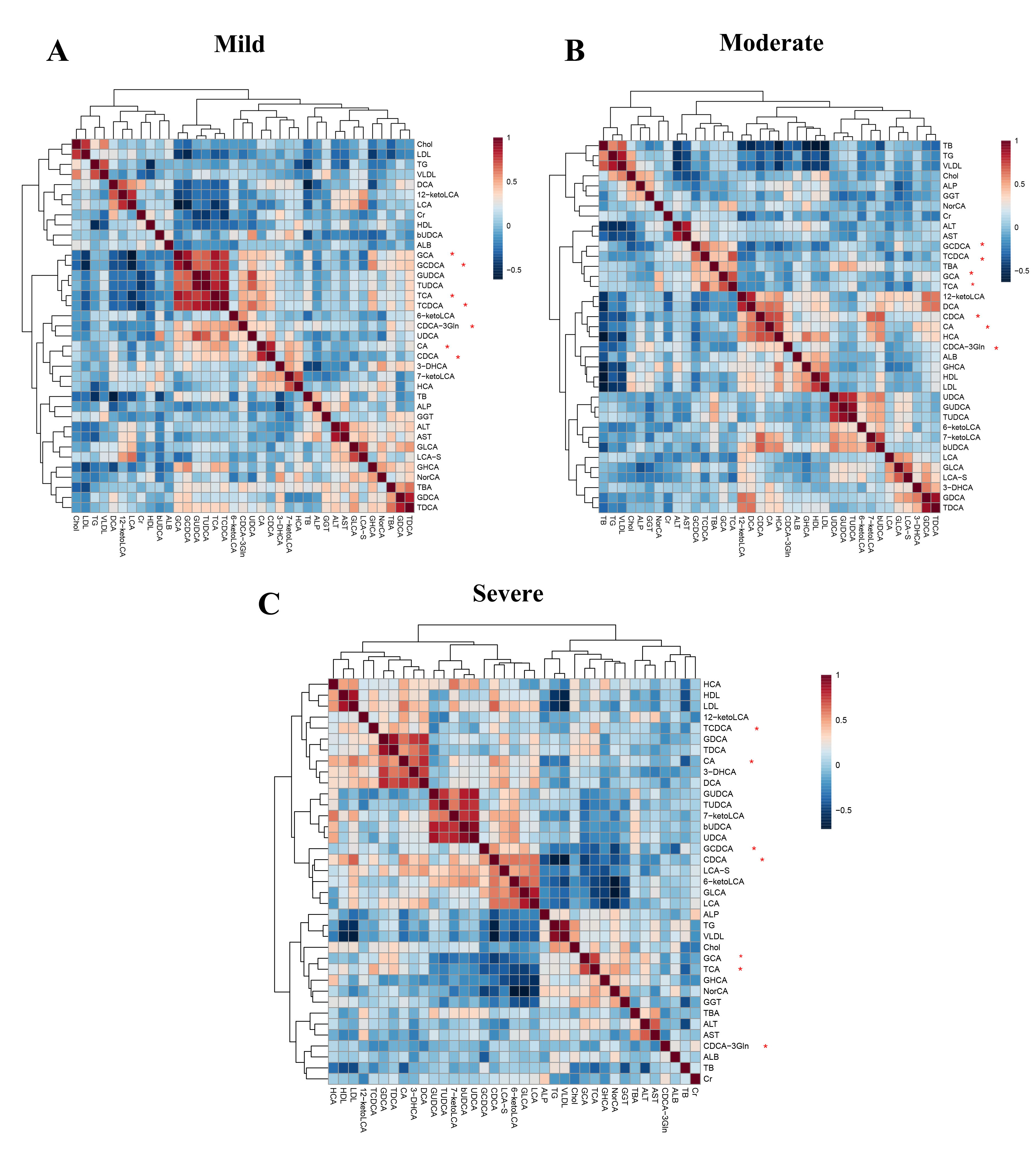

Supplement: Supplementary file 1 [file metabolites-11-00852-s001.zip › Figure S3.tif]

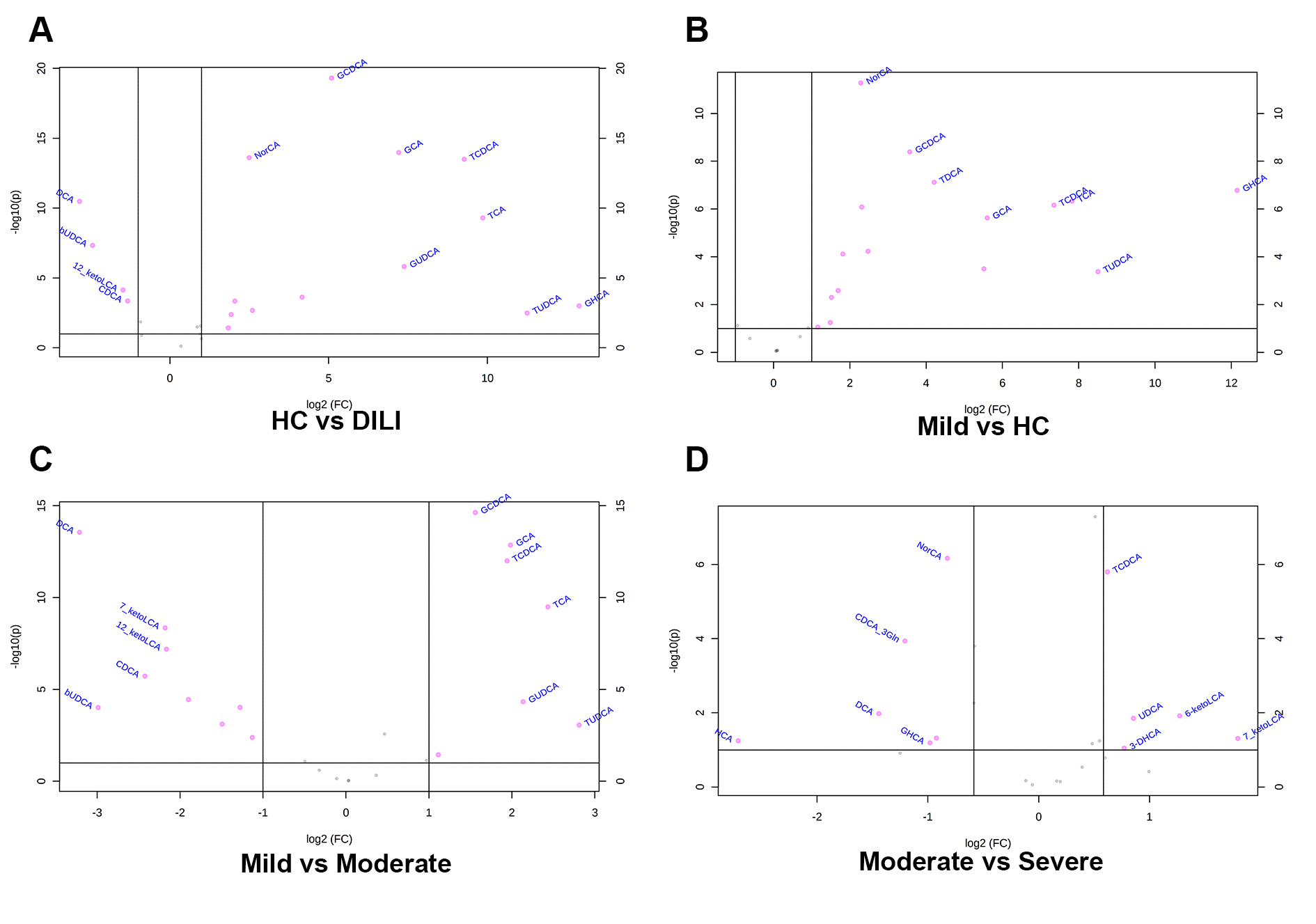

Supplement: Supplementary file 1 [file metabolites-11-00852-s001.zip › Figure S4.tif]
